# The Effect of Beta-Carotene on Cognitive Function: A Systematic Review

**DOI:** 10.3390/brainsci13101468

**Published:** 2023-10-17

**Authors:** Diana Marisol Abrego-Guandique, Maria Luisa Bonet, Maria Cristina Caroleo, Roberto Cannataro, Paola Tucci, Joan Ribot, Erika Cione

**Affiliations:** 1 Department of Health Sciences, University of Magna Graecia Catanzaro, 88100 Catanzaro, Italy; dianamarisol.abregoguandique@unicz.it (D.M.A.-G.); mariacristina.caroleo@unicz.it (M.C.C.); 2 Galascreen Laboratories, University of Calabria, 87036 Rende, Italy; r.cannataro@gmail.com; 3 Laboratory of Molecular Biology, Nutrition, and Biotechnology (LBNB), Universitat de les Illes Balears, 07122 Palma, Spain; luisabonet@uib.es (M.L.B.); joan.ribot@uib.es (J.R.); 4 Institut d’Investigació Sanitària Illes Balears (IdISBa), 07120 Palma, Spain; 5 CIBER de Fisiopatología de la Obesidad y Nutrición (CIBERobn), 07122 Palma, Spain; 6 Research Division, Dynamical Business & Science Society, DBSS International SAS, Bogota 110311, Colombia; 7 Department of Pharmacy, Health and Nutritional Sciences, University of Calabria, 87036 Rende, Italy; paola.tucci@unical.it

**Keywords:** cognitive function, β-carotene, diet supplements, synergic effect

## Abstract

β-carotene is a powerful antioxidant and dietary precursor of vitamin A whose role in maintaining mental health and cognitive performance, either alone or in combination with other dietary compounds, has been a topic of recent research. However, its effectiveness is still unclear. This systematic review, conducted according to the PRISMA guideline and assisted by the MySLR platform, addressed this issue. A total of 16 eligible original research articles were identified. Dietary intake or β-carotene serum levels were associated with improved measures of cognitive function in 7 out of 10 epidemiological studies included. In intervention studies, β-carotene consumption alone did not promote better cognitive function in the short term, but only in a long-term intervention with a mean duration of 18 years. However, all but one intervention study suggested the beneficial effects of β-carotene supplementation at doses ranging from 6 mg to 50 mg per day in combination with a multicomplex such as vitamin E, vitamin C, zinc, or selenium for a period of 16 weeks to 20 years. Despite the current limitations, the available evidence suggests a potential association between β-carotene dietary/supplementary intake and the maintenance of cognitive function. The β-carotene most probably does not act alone but in synergy with other micronutrients.

## 1. Introduction

Cognitive functions are mental domains that enable us to receive, process, and elaborate information. These functions include complex attention, executive function, language, learning and memory, perceptual-motor function, and social cognition. There is continuous coordination and switching between memory, attention, and executive function sub-processes [1]. Cognitive dysfunction can be defined as a disruption or lack of equilibrium within the brain’s structural and functional organization at distinct levels: the molecular/cellular level, the level of local circuits, and the level of large-scale networks that involve neural interactions, protein–protein interaction networks, and gene–miRNA interactions [2]. miRNAs have potential as biomarkers [3] and can be associated with poor nutritional status [4].

Cognitive behaviors are crucial for individuals’ overall health and well-being regardless of their specific condition. Maintaining optimal cognitive function is essential for daily functioning, learning, work performance, and overall quality of life [5]. Cognitive function is vital for athletes’ sports performance because it affects their decision-making skills and reaction time. It may be more relevant in open sports that require constant attention or adaptation to changing situations [6,7].

Cognitive function evolves throughout life. The likelihood of experiencing mild cognitive impairment increases with age [8]. As people live longer, cognitive impairment is becoming more prevalent, posing a significant public health challenge. The prevalence of cognitive impairment is estimated to be 19% for those under 75 years old and 29% for those over 85. In addition, 30% of people over 65 are diagnosed with Alzheimer’s [9]. With the rise in the number of older adults, these numbers are expected to increase. Additionally, chemotherapy-induced cognitive impairment, often called “chemobrain”, can affect cancer patients during and after treatment [10,11]. Moreover, many conditions are associated with brain fog, a nebulous concept that eluded scientific examination until the COVID-19 outbreak put it under the spotlight [12,13,14]. Therefore, it is crucial to develop strategies to combat cognitive decline, with prevention by diet changes or supplementation seen as a possible strategy.

There is evidence that oxidative damage contributes to age-related cognitive decline [15]. Among the leading causes of cognitive decline, the increase in oxidative stress in the aging brain is widely documented in humans [16]. Oxidative stress also underlies chemotherapy-induced mild cognitive impairment. Chemotherapy causes lipid peroxidation, depleting cellular levels of antioxidant molecules such as minerals and vitamins, which in turn generates free radicals contributing to oxidative stress [17].

Considering that carotenoids act as antioxidants and anti-inflammatory agents [18], their intake in the diet could be a prevention strategy to maintain and improve cognitive health. No specific recommendations regarding the intake of carotenoids have been issued to date. The total carotenoid intake in European countries (median values) ranges from ~9.5 to 16 mg/d (~3 to 6 mg/d for β-carotene) [19]. β-carotene is the most important natural carotenoid and the primary dietary source of pro-vitamin A. It is known for its antioxidant properties and free radical scavenging actions due to abundant unsaturated bonds in its molecule. It contributes to about 30–35% of the dietary intake of vitamin A in Western countries, while in developing countries it is the most important source of vitamin A [20]. β-carotene is found in vegetables, fruits, and soup/bouillon [21] and it is sometimes used as a food coloring additive. The importance of vitamin A spans from vision to sustaining immunity and fertility [22,23,24]. Therefore, it is important to understand the impact of β-carotene on cognitive function and consider its action, either alone or in combination with other bioactive compounds, for future intervention in this specific area.

We here conducted a systematic review of studies combining psychological approaches and nutrition with the aim of evaluating the impact of β-carotene on cognitive domains in adults. The review was facilitated by a semi-automated tool that, after the identification and loading of databases on the platform, selects and evaluates quality contributions and analyzes and synthesizes the results. In particular, we used the MySLR digital platform, a digital tool that implements the Latent Dirichlet Allocation (LDA) algorithm [25] to analyze a large number of scientific publications using text mining. To date, findings regarding the possibility that dietary intake or supplementation with β-carotene and interaction may delay the onset of cognitive decline or even ameliorate cognitive performance remain inconsistent across studies. There is currently no published systematic review or meta-analysis to our knowledge that provides a quantitative measure of the association between β-carotene and cognitive function. Therefore, a systematic review was conducted to evaluate the potential clinical effects of β-carotene intake on cognition in adults.

## 2. Methods

The systematic review was conducted according to the Preferred Reporting Items for Systematic Reviews and Meta-Analyses (PRISMA) guidelines [26] and registered at the PROSPERO International Prospective Registry (CRD42023422784). The methodological approach is based on three steps: (i) paper location and selection, (ii) paper analysis, and (iii) results presentation. We adopted a semi-automated approach using the MySLR platform upon registration (available at https://myslr.unical.it, accessed on 31 May 2023). This digital tool reproduces “human-like intelligence” as closely as possible by implementing the LDA algorithm. We loaded the papers on MySLR to offer a complete and exhaustive overview of scientific research.

### 2.1. Paper Location and Selection

Two investigators (D.M.A-G. and E.C.) independently conducted the searches on the PubMed, Scopus, and Web of Science databases to identify publications in peer-reviewed journals published before 31 May 2023. The search was conducted using the Boolean operators “AND” and “OR” to combine the following terms: (“Cognition” OR “Cognitive function” OR “Cognitive Dysfunction” OR “Cognitive decline” OR “Cognitive Outcomes” OR “Cognitive impairment” OR “Mild cognitive impairment”) AND (“Memory” OR “Attention” OR “Executive Function” OR “Working Memory” OR “processing speed” OR “visuospatial” OR “verbal fluency” OR “word fluency” OR “Learning” OR “Thinking”) AND (“beta carotene” OR “beta-carotene” OR “beta-carotene” OR “βcarotene” OR “β-carotene” OR “β carotene”).

### 2.2. Study Selection and Data Extraction

Studies were included in the systematic review that assessed the effect of β-carotene alone or in combination with other compounds on cognitive function, which were all published before 31 May 2023. The inclusion criteria for this systematic review were as follows:

*Population*: studies in humans conducted with participants ≥18 years of age without mental disorders. 

*Intervention*: studies that reported the β-carotene content of foods or dose-containing supplements or studies that reported blood levels of β-carotene.

*Outcomes*: studies that provided sufficient information about cognitive outcomes.

*Types of Study*: randomized clinical trials or prospective (cross-sectional study) or longitudinal study. 

Articles were excluded from the systematic review for the following reasons: studies not published in English; reviews, meta-analysis, letters, conference papers, comments, or book chapters; studies on animal models or in vitro experiments.

Disagreements were resolved through discussion in order to reach a consensus or by means of a third reviewer (M.L.B. and M.C.C.)

Data from all included articles were extracted by one author (D.M.A-G.) and checked by two authors (E.C. and M.L.B.). The following information was recorded: authors’ names, publication year, study country, study design, participant characteristics (sample size, gender, and age), dietary intake/intervention/blood levels, outcomes of interest, cognitive tests used, and results. The levels of evidence within each condition are evaluated using a levels of evidence framework for quantitative research on intervention studies that was adapted from Melnyk and Fineout-Overholt [27].

### 2.3. Quality Assessment and Levels of Evidence

The possibility of bias in the design and analysis of each study was assessed by two different evaluators (D.M.A-G. and E.C.) using the NIH Study Quality Assessment Tool https://www.nhlbi.nih.gov/health-topics/study-quality-assessment-tools (accessed on 10 June 2023) and three more reviewers were consulted when necessary (R.C., P.T., and J.R.). Specifically, the Quality Assessment Tool for Observational Cohort and Cross-sectional Studies and the Quality Assessment of Controlled Intervention Studies were used; both forms have 14 questions designed to help focus on the key concepts for evaluating the internal validity of a study, such as the risk of potential selection, method, information, measurement, and confounding bias.

### 2.4. Results Presentation

The final stage of the methodological approach is explained in the sections “Results” and “Discussion.” This step aims to comprehensively define and analyze the outcomes obtained from the MySLR procedure. It involves meticulously examining the relevant literature to describe and discuss the results clearly.

## 3. Results

**Figure 1:**
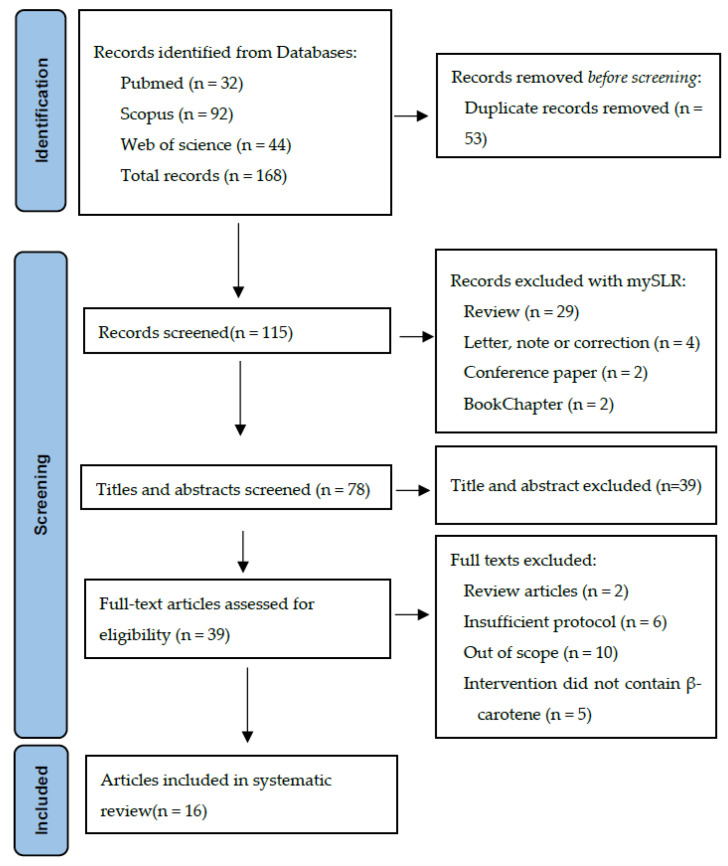
PRISMA flow diagram showing the algorithm for selecting eligible studies.

A total of 168 records were identified from the initial literature search of the three databases (PubMed, Scopus, and Web of Science). After excluding the duplicate records, 115 items remained. Of these, MySLR removed 37 papers because they were reviews, book chapters, meta-analyses, or other irrelevant publications for our systematic review. Of the 78 studies remaining, 39 were discarded based on their abstracts. After full-text reading and analysis, 23 records were further excluded for failing to meet the inclusion/exclusion criteria. Therefore, a total of 16 studies were considered eligible. The PRISMA flowchart in Figure 1 shows the selection of the studies for this systematic review.

**Figure 2:**
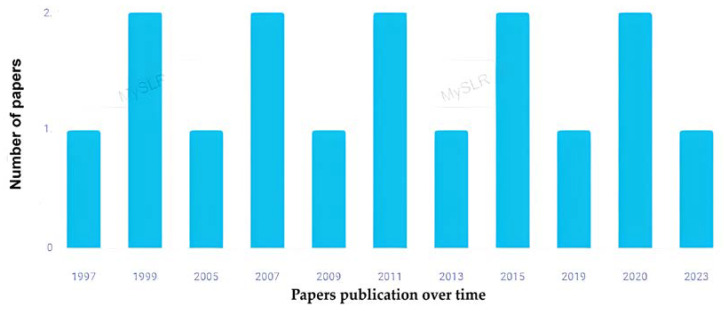
Distribution by year of the studies on the relationship between β-carotene and cognitive function included in the systematic review (*n* = 16) (created with MySLR).

Epidemiological studies on the relationship between β-carotene and cognitive function have not shown a regular trend over time, as shown in Figure 2. The first study was published in 1997 [28]. Four scientific articles were published in the last five years [29,30,31,32].

### 3.1. Characteristics of the Studies Included

Table 1 summarizes the characteristics of the studies reporting dietary β-carotene or β-carotene supplementation, while Table 2 summarizes those of the studies reporting blood levels of β-carotene. Articles in both tables appear in descending order by year of publication.

Of the 16 eligible studies, 4 were prospective studies, 5 were cross-sectional studies, and 7 were randomized intervention studies. The studies were published from 1997 to 2023 and conducted in seven countries: the United States, Germany, France, Switzerland, the United Kingdom, China, and the Netherlands. Nine studies were conducted in both sexes [28,29,33,34,35,36,37,38,39], whereas two studies were exclusively conducted in males [30,40] and five in females [31,32,41,42,43]. The ages of all the participants ranged from 30 to 100 years (mean 53.2 ± 13.6).

All studies measured cognitive function in healthy subjects, except for two studies focused on participants affected by low cognitive function [29] and mild cognitive impairment [32] and one study in women with cardiovascular disease [42].

**Table 1:** Studies reporting dietary β-carotene or supplementation.

Study	Location	Level of Evidence	Study Design	Participant Characteristics	Dietary Intake/Intervention	Conditions	Outcome of Interest	Cognitive Test Used	Reference
Zhong et al. (2023)	USA	4	Cross-sectional	*n* = 2009; 985 males and 1024 females; aged >60 y	Dietary BC intake: Q1 (≤338 μg/d); Q2 (338 to ≤819 μg/d); Q3 (819 to ≤2222.5 μg/d); Q4 (>2222.5 μg/d)	Participants in NHANES 2011–2014 older than 60 y that had completed data on cognitive function	Memory Processing speed and working memory Categorical verbal fluency	CERAD WL DSST AFT	[29]
Beydoun et al. (2020)	USA	3	Prospective cohort	*n* = 1251; male; aged 30–65 y at baseline	Dietary BC intake: overall, 1819 ± 2882 μg/d; T1 = 309 ± 289 μg/d; T2 = 1226 ± 987 μg/d; T3 = 3980 ± 4152 μg/d	Healthy subjects; BC intake assessed at V1; cognitive performance assessed at V1 (2004–2009) and V2 (2009–2013)	Global cognition Attention Learning/memory Executive function Visuo-spatial/visuo-construction ability Psychomotor speed Language/verbal	MMSE CVLT Digit span test BVRT AFT BTA TMT A/B CDT	[30]
Yuan et al. (2020)	USA	3	Prospective cohort	*n* = 49493; female (nurses); mean age 48 y	Dietary BC intake: Q1 = 2.5 ± 0.5 mg/d; without supplements 2.3 ± 5.4 mg/d; Q5 = 8.6 ± 2.1 mg/d; without supplements 6.9 ± 2.1 mg/d	Healthy subjects; FFQs collected at baseline and periodically until 2006; self-reported SCF in 2012 and 2014	General memory Executive function Attention Visuospatial skills	Assessment of SCF based on 7 yes/no questions on recent changes	[31]
Li et al. (2015)	China	2	Intervention	*n* = 276; 116 male and 160 female; aged 67.06 ± 5.33 y	Groups A, B, C, and D received 200 mg/d VE and 300 mg/d VC, combined with 16.7, 8.4, 5.6, or 0 mg/d BC, respectively. Group E: 5 mg/d VE	Healthy subjects; 5 groups A-E (*n* = 60 per group, there were 24 drop outs); 16 weeks of intervention; cognitive function assessed prior to and after the intervention	Global cognitive function	MMSE HDS	[33]
Nooyens et al. (2015)	Netherland	3	Prospective cohort	*n* = 2613; both sexes; aged 43–70 y at baseline	Dietary intake at baseline and follow-up through a validated self-administered semi-quantitative FFQ. Estimated BC intake: 1480 ± 593 μg/d	Healthy subjects; cognitive performance assessed 5 y after baseline	Global cognitive function Memory Processing speed Cognitive flexibility	15 Words Learning test Stroop test WFT Letter digit substitution test	[34]
Kesse-Guyot et al. (2011)	France	2	Randomized double-blind placebo-controlled trial	*n* = 4447; both sexes; aged 45–60 y	Multivitamin (6 mg/d BC) or placebo	Healthy subjects; 8 y of intervention; cognitive performance assessed 6 y after the end of the intervention	Episodic memory Executive function Verbal memory Verbal fluency	TMT FDST RI-48 Semantic fluency Phonemic fluency	[37]
Péneau et al. (2011)	France	3	Prospective cohort	*n* = 2533; male and female; aged 45–60 y at baseline	Dietary intake of FVs grouped based on their nutrient content: folate-rich FVs and BC-rich FVs	Healthy subjects; cognitive performance assessed 13 y after baseline	Episodic memory Lexical semantic memory Mental flexibility Working memory	RI-48 Verbal fluency test TMT FDST	[38]
Kang et al. (2009)	USA	2	2 × 2 × 2 randomized placebo-controlled trial	*n* = 2824; female; aged >65 y	BC (50 mg every other day) or placebo alone or combined with VE, VC, or both	Women with cardiovascular disease; 3.5 y of intervention	Global cognition Verbal memory Category fluency	TICS EBMT CFT	[42]
Grodstein et al. (2007)	USA	2	Randomized double-blind placebo-controlled trial	*n* = 4052; male; mean age 55.9 y	50 mg BC on alternate days	Healthy subjects; 18 y of intervention	Global cognition Verbal memory Category fluency	TICS EBMT CFT	[40]
Wolters et al. (2005)	Germany	2	Randomized double-blind placebo-controlled trial	*n* = 220; female; aged 60–91 y	Multivitamin (9 mg/d BC) or placebo	Healthy subjects, free-living women; 6 months of intervention	Total Intellectual Quotient Intelligence Assessment of mild up to severe memory disorders	WAIS-III KAI BAT	[41]
Smith et al. (1999)	UK	2	Randomized double-blind placebo-controlled trial	*n* = 205; male and female; aged 60–80 y	Multivitamin (12 mg/d BC) or placebo	12 months of intervention	Episodic memory Psychomotor speed Attention	NART score CFQ score	[39]

T, tertile; Q, quintile; V, visit; VC, vitamin C; VE, vitamin E. Other abbreviations are presented in the Abbreviations section.

**Table 2:** Studies reporting serum levels of β-carotene.

Study	Location	Level of Evidence	Study Design	Participant Characteristics	Blood Levels	Condition	Outcome of Interest	Cognitive Test Used	Reference
Gerger et al. (2019)	Germany	2	Multi-centered randomized controlled trial	*n* = 56; female; aged 73.1 ± 5,8 y	BC (μM): 0.74 ± 0.65	Subjects with mild cognitive impairment participating in the NeuroExercise study	Global cognition Verbal memory Working memory Attention Executive function	MoCA ISLT ONB TMT	[32]
Johnson et al. (2013)	USA(Georgia)	4	Cross-sectional	*n* = 298, of which 78 were octogenarians and 220 centenarians; male and female	BC (nmol/L): octogenarians, 568 ± 855; centenarians, 460 ± 432	Institutionalized and community-dwelling subjects compared; study conducted from 2001 to 2009	Memory processing speed Attention Executive functioning	MMSE GDRS SIB FOME WAIS-III Similarities subtest BDS COWAT	[35]
Akbaraly et al. (2007)	France	4	Cross-sectional	*n* = 589; 361 female and 228 male; aged 73.5 ± 3 y	*Trans*-BC levels, 0.73 ± 0.52 μmol/L; *cis*-BC levels, 0.10 ± 0.12 μmol/L	Healthy old subjects	Global cognition Motor speed Working memory Executive function Attention and logical reasoning Verbal fluency	MMSE TMTA TMTB DSST WFT	[36]
Perkins et al. (1999)	USA	4	Cross-sectional	*n* = 4809; female; aged >60 y	BC normalized on units of total cholesterol: <0.06; 0.06–0.09; 0.09–0.15; >0.15	Elderly, multi-ethnic group followed from 1988 to 1994	Memory	Assessed using delayed recall (6 points from a story and 3 words), with poor memory defined by a combined score < 4	[43]
Perrig et al. (1997)	Switzerland	4	Longitudinal and cross-sectional comparisons	*n* = 442; 312 male, 132 female; aged 65 to 94 y	BC measured in 1971 (T1) and 1993 (T2); BC levels (µg/dL): T1, 0.51 ± 0.31; T2, 0.72 ± 0.48	Healthy old subjects selected in T2 by random sampling from a large cohort established in T1; cognitive function assessed in T2	Implicit and explicit memory Working memory Semantic memory	Free recall and recognitionWAIS-R Vocabulary test	[28]

T, time. Other abbreviations are presented in the Abbreviations section.

**Figure 3:**
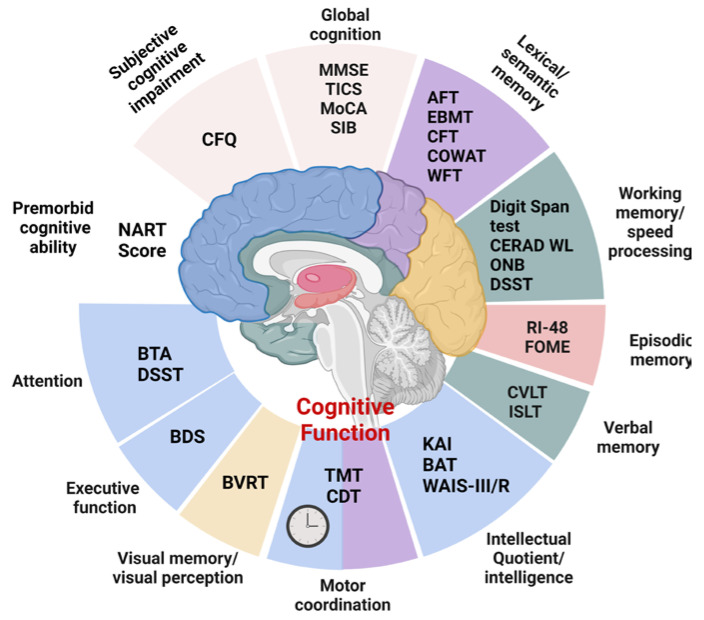
Scheme of the cognitive test batteries and assessment tools used in the systematically revised literature. ***Global cognition*** was assessed with the following: **MMSE:** mini-mental state examination; **TICS**: Telephone Interview of Cognitive Status; **MoCA:** Montreal Cognitive Assessment; **SIB**: Severe Impairment Battery. ***Lexical/semantic memory*** was assessed with the following: **AFT**: Animal Fluency Test; **EBMT**: East Boston Memory Test; **CFT**: Category Fluency Test; **COWAT**: Controlled Oral Word Association Test; **WFT**: Word Fluency Test. ***Working memory/speed processing*** was assessed with the following: the digit span test; **CERAD WL**: Consortium to Establish a Registry for Alzheimer’s Disease Word Learning; **ONB**: One Back Task; **DSST**: Digit Symbol Substitution Test. ***Episodic memory*** was assessed with the following: **RI-48**: Cued Recall Test; **FOME**: Fuld Object Memory Evaluation. ***Verbal memory*** was assessed with the following: **CVLT**: California Verbal Learning Test; **ISLT**: International Shopping List Task. ***Intellectual quotient/intelligence*** was assessed with the following: **KAI**: Kurztest fuer Allgemeine Intelligenz; **BAT**: Berliner Amnesie Test; **WAIS-III**: Wechsler Adult Intelligence Scale Revised. ***Motor coordination*** was assessed with the following: **TMT**, Trail Making Test; **CDT**: Clock Drawing Test. ***Visual memory/visual perception*** was assessed with the **BVRT**: Benton Visual Retention Test. ***Executive function*** was assessed with the **BDS**: Behavioral Dyscontrol Scale. **Attention** was assessed with the following: **BTA**: Brief Test of Attention; **DSST**: Digit Symbol Substitution Test. ***Premorbid cognitive ability*** was assessed with the **NART**: National Adult Reading Test. ***Subjective cognitive impairment*** was assessed with the **CFQ**: Cognitive Failures Questionnaire. The visualization was created with BioRender.

The systematic review included studies that utilized a wide variety of cognitive test batteries (Figure 3), such as the MMSE [30,33,35,36], TICS [40,42], MoCA [32], and SIB [35] for global cognition. Memory was assessed at different levels (lexical and semantic memory, through verbal fluency tests [34,38], the EBMT [40,42], the CFT [29,40,42], the COWAT [35], and the WFT [34,36]; working memory and speed processing, through the digit span test which includes the FDST [30,37,38], CERAD WL [29], ONB [32], and DSST [29,36]; episodic memory, with RI-48 [37,38] and FOME [30]; and verbal memory, with the CVLT [30] and ISLT [32]), whereas visual memory/visual perception was evaluated using the BVRT [30]. The KAI, BAT, and WAIS-III/R tests were administered for Total Intellectual Quotient and Intelligence [28,35,41]. The relationship between motor coordination and executive function was evaluated using the TMT [30,32,36,37,38], while the CDT was used for executive function practice–constructive skills and visuospatial abilities [30]. Subjective cognitive impairment was measured by the CFQ, which is a global recognized method, while for premorbid cognitive ability the NART score was used [39]. Finally, attention was measured using the BTA [30] and the DSST [36].

Grodstein et al. carried out the most important and prolonged intervention study with β-carotene alone, with a mean treatment period of 18 years [40]. Five studies assessed daily dietary intake including β-carotene using food frequency questionnaires [29,30,31,34,38], and five studies used multivitamin supplementation (controlled dosage of β-carotene) [33,41], of which three used a placebo [37,39,42].

The MySLR platform generates synthesis results. We adjusted the number of topics to be extracted (k value) to two, which ensured a satisfactory value of topic coherence (−1.25) with easy interpretation of the results [25]. The MySLR platform allowed us to use the LDA algorithm, which identifies the most relevant words for each topic, creating a word cloud. The words “vitamin” and “supplementation” received the higher scores in Topic 1; while the word “antioxidant” received the second highest score in Topic 2 after “vitamin”.

### 3.2. Risk of Bias Assessment

**Figure 4:**
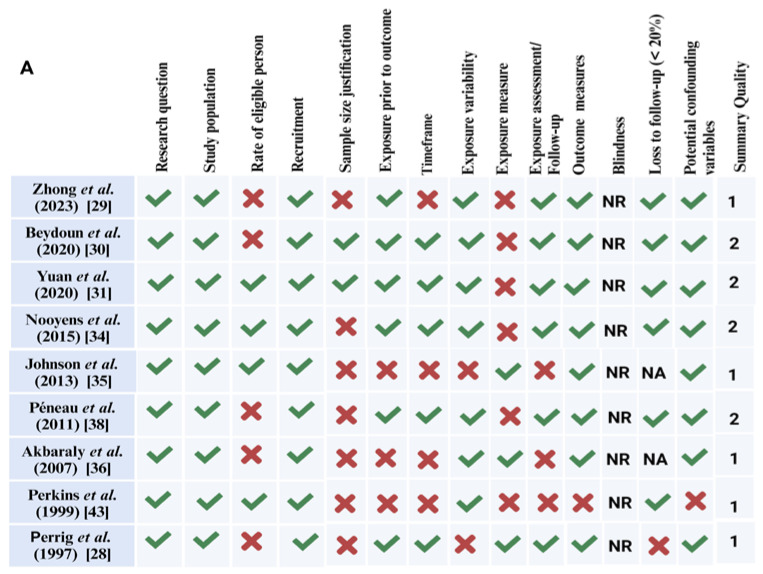

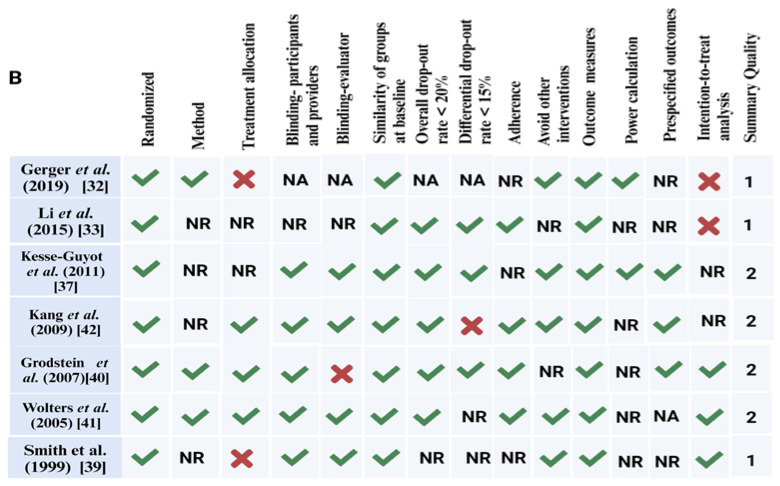
(**A**) Summary of risk-of-bias assessment according to the National Institutes of Health Quality Assessment Tool for Observational Cohort and Cross-Sectional Studies (NIH, 2014) [44]. (**B**) Summary Quality Assessment according to Health Quality Controlled Intervention Studies (NIH, 2014). The quality rating is 0 for **poor** (0–4 out of 14 questions), 1 for **fair** (5–9 out of 14 questions), or 2 for **good** (>10 out of 14 questions) [28,29,30,31,32,33,34,35,36,37,38,39,40,41,42,43]. NA: not applicable, NR: not reported; created with BioRender.

The risk of bias assessment is summarized in Figure 4A,B. As mentioned above, we used two tools: the Quality Assessment Tool for Observational Cohort and Cross-sectional Studies (*n* = 9) and Quality Assessment of Controlled Intervention Studies (*n* = 7). Using these tools, out of the 16 studies included in the systematic review, 8 studies were rated as “good” [30,31,34,37,38,40,41,42] and 8 as “fair” [28,29,32,33,35,36,39,43].

### 3.3. Topic Identification

The LDA algorithm allowed us to identify two topics regarding β-carotene and cognitive function in older adults, which are presented and discussed in this section. We developed the discussion starting from Topic 2 since it deals with the impact of dietary β-carotene intake, and we then treated Topic 1, which is more specific as it examines the influence of supplementation of β-carotene, more generally as part of multivitamin formulations.

#### 3.3.1. Topic 2: Antioxidants: β-carotene Dietary Intake and Its Relationship with Cognitive Function

By examining the top 30 most significant terms and their frequency within the ten papers categorized under this topic [28,29,30,31,32,34,35,36,38,43] and further analyzing them, it became apparent that the fundamental aspect of Topic 2 was the relationship between cognitive function and the dietary intake or serum levels of β-carotene. The concentration of carotenoids in serum is considered to reflect short-term dietary intake and it is widely accepted as a good biomarker of fruit and vegetable intake [45,46].

Studies clustered in Topic 2 and their main results are summarized in Table 3. These studies aim to understand whether higher consumption of β-carotene-rich foods such as vegetables and fruits or increased serum levels of β-carotene are associated with improved cognitive function. It is currently hypothesized that consuming fruits and vegetables can protect against age-related cognitive impairments. It is probable that by increasing the intake of dietary antioxidants such as β-carotene, the damaging effects of free radicals on neurons can be slowed down, potentially protecting against cognitive decline and conditions such as dementia. Modifying antioxidant intake through supplementation or dietary changes is relatively easy to implement, making it an attractive strategy.

#### 3.3.2. Topic 1. The Impact of β-carotene Supplementation with a Multivitamin on Cognitive Performance

The other topic we have identified is the effect of β-carotene supplementation on cognitive performance. Supplementation with β-carotene as a single molecule is rarely studied because it is usually studied in synergy with other antioxidant molecules or vitamins.

Studies clustered in Topic 1 and their main results are summarized in Table 4. These studies monitored the effect of β-carotene supplementation on various aspects of cognitive function, including memory, attention, and executive function. Only one study focused on evaluation of the effects of supplementation of β-carotene as a single molecule [40]. The other studies used controlled dosages of β-carotene in combination with multivitamins or minerals. Interestingly, one study evaluated the blood levels of β-carotene during supplementation [37].

## 4. Discussion

The purpose of the present review was to summarize the available clinical evidence regarding the potential use of β-carotene intake as a nutritional strategy for cognitive maintenance. To date, few clinical trials have investigated the relationship between β-carotene and cognition, and epidemiological studies examining the association between dietary and supplementary β-carotene intake and cognitive function have yielded varying and inconclusive results. Here, we extrapolate that dietary intake and β-carotene serum levels have been associated with measures of cognitive function in some studies [28,29,31,32,35,38], but not in all of them [34,36,43]. In particular, Zhong et al., using NHANES data, reported that β-carotene dietary intake was inversely associated with cognitive function decline. There was an approximately linear dose–response relationship between β-carotene dietary intake and CERAD WL, AFT, and DSST test results; moreover, they reported differences in cognitive function between sexes [29]. It is important to underlay here that these batteries of cognitive function tests have been widely used in large epidemiological and clinical studies [47,48]. Their validity in clinical assessment was determined in neuropathological patients and, for CERAD WL, also confirmed post-mortem by brain autopsy in a group of 176 primary dementing illness patients [48]. Similarly, the study of Bacchetti et al. observed sex differences in plasma β-carotene levels, suggesting that the protective effect of β-carotene on cognitive decline is higher in women than in men [49]. Two other studies have shown that the association between β-carotene and ISLT has good sensitivity to verbal memory alteration [28,32]. It has been demonstrated that the ISLT is sensitive to verbal memory deficits, has the potential to be adapted to cultural groups, and allows for accurate comparison [50]. In particular, Perrig and co-workers found a positive correlation between elevated levels of β-carotene in blood and improved semantic memory performance according to the ISLT and concluded that β-carotene serum levels remain a significant predictor of semantic memory performance [28]. Johnson et al. examined the influence of plasma β-carotene, which correlated positively with most measures of cognitive function [35]. In accordance, a lower consumption of β-carotene was linked in the Rotterdam study to decreased cognitive performance as assessed by the MMSE [51]. In addition, Yuan et al. described that long-term greater dietary intake of total β-carotene was associated with lower odds of moderate and poor subjective cognitive function [31]. 

Besides this epidemiological evidence, the findings of several randomized clinical trials support the protective effect of β-carotene against cognitive impairment. In the first, the Physicians’ Health Study (PHS), men treated with 50 mg of β-carotene on alternate days over the course of 18 years showed higher overall scores in terms of verbal and cognitive memory than those treated with placebo. However, there was no effect in men who underwent a single year of supplementation [40]. These findings show that the purported cognitive benefits of β-carotene supplementation may be related to earlier age or a longer duration of exposure. In the second study, indicated as the SU.VI.MAX study, higher episodic memory and semantic fluency test scores were reported alongside supplementation with various antioxidants, namely VC, VE, and β-carotene, particularly among non-smokers and subjects with low serum concentrations of antioxidants at baseline. However, the independent contribution of β-carotene to the general observed effects in this study cannot be determined [37]. In agreement with these previous findings, the supplementation strategy of VE and VC combined with β-carotene significantly improved cognitive function in the elderly subjects, particularly with higher doses of β-carotene [33]. In a randomized trial in women with cardiovascular disease, β-carotene treatment showed no effect on cognitive decline after 10 years, although β-carotene-containing supplements were effective in slowing cognitive decline in a subgroup of women who consumed little β-carotene in their diet [42]. 

On the contrary, higher intakes of FVs, vegetables alone, and β-carotene-rich FVs were associated with poorer executive function and poor performance on the FDST [38]; this inverse association could be linked to the presence of pesticides in vegetables, as exposure to pesticides elevates the risk of cognitive impairment [52]. We also reported the study of Beydoun et al. in which dietary β-carotene intake was associated with faster cognitive decline and poorer performance on the CDT [30]. Finally, in two studies, the supplementation of multivitamin complexes containing β-carotene at 9 mg (for six months) and 12 mg (for one year) per capsule, respectively, had no effect on cognitive performance [39,41]. 

The benefits of β-carotene on cognitive function, as found in a significant part of the studies reviewed herein, could be explained by several non-mutually exclusive biochemical mechanisms, starting with the antioxidant activity of this compound. β-carotene is considered a powerful chelator of singlet oxygen and reacts with several species of free radicals. The conjugated double chain is responsible for eliminating singlet oxygen, and the greater the number of conjugated bonds, the greater the ability to eliminate singlet oxygen [53]. It has also been noted that β-carotene and other antioxidant substances, such as vitamin E, can function synergistically; in fact, studies suggest that low serum levels and low intake of tocopherols and tocotrienols are associated with the risk of cognitive impairment in older adults, which reinforces the hypothesis that vitamin E in its different forms plays a role in the maintenance of cognitive function in aging [54]. At the same time, β-carotene and vitamin E work together to prevent lipid peroxidation [55]. However, in some conditions, including high doses of antioxidant intake or the oxidative status of smokers, β-carotene may increase lipid peroxidation and DNA oxidative damage. β-carotene is sensitive to degradation and oxidation, and under certain conditions it can act as a pro-oxidant [56], favoring the production of reactive oxygen species (ROS) such as epoxides and carbonyls [57]. 

Additionally, β-carotene intake could help to mitigate brain fog or cognitive impairment through its effect on calcium/calmodulin-dependent protein kinase IV (CAMKIV). This enzyme belongs to the Ser/Thr kinase family. In the brain, it is found in the cerebellar cortical granules. CAMKIV plays a role in angiogenesis, the inhibition of apoptosis, and cell signaling in a calcium-dependent manner. At elevated intracellular calcium ion concentration, CAMKIV forms Ca^2+^/calmodulin complexes and induces the phosphorylation of transcription factors. CAMKIV is considered an important factor in neurodegenerative disorders, as well as in several types of cancer [58]. It has been shown that β-carotene, by itself, can bind to the active site of CAMKIV with high affinity, forming a stable complex which, in turn, results in decreased CAMKIV activity [59]. This ability could make β-carotene an attractive supplement in neurodegenerative disorders, and also in cancer treatment, since it has shown no cytotoxic effects in vitro [59,60]. Interestingly, Kim et al. showed that β-carotene is associated with increased levels of Brain-Derived Neurotrophic Factor (BDNF) in animal models [61]. BDNF functions in the brain to regulate synapses, exhibiting structural and functional effects on excitatory or inhibitory synapses [62], and is a key molecule in plastic changes related to learning and memory [63].

Finally, there is also evidence to support the role of β-carotene in favorably influencing cognition through its role as a vitamin A precursor. The conversion of vitamin A to retinoic acid, which activates retinoic acid receptors, occurs more quickly in the brain than in other target organs. Synaptic plasticity in areas of the brain involved in learning and memory, such as the hippocampus, is controlled by the controlled synthesis of retinoic acid [64]. Studies in rodents have shown that retinoic acid induces hippocampal neurogenesis and neuronal differentiation [65], whereas vitamin A deficiency decreases hippocampal plasticity and increases amyloid β deposition in rodent models [66]. 

The results of the current systematic review have been mixed, with a majority of studies reporting positive effects on cognitive performance while others show no significant improvement or even a negative impact. The disparity of results could be due to differences in the features of study populations. It is necessary to enhance our knowledge of the impact of age and gender on β-carotene absorption and its conversion to vitamin A, as well as to comprehend the effects of short-term versus long-term supplementation and the influence of single nucleotide polymorphisms (SNPs), such as in cognitive function-related genes and carotenoid retinoid metabolism-related genes. β-carotene may provide some form of defense against cognitive aging in people who are more genetically predisposed to it, as shown for the APOε 4 allele [67]. It is also worth mentioning that many neurodegenerative disorders, including Alzheimer’s disease, often display coexistence with metabolic dysfunctions and that ablation of the β,β-Carotene-9’,10’-oxygenase 2 (BCO2) enzyme that catalyzes the asymmetric cleavage of carotenoids remodels the hypothalamic metabolome, leading to metabolic disorders in mice [66].

β-carotene could also provide such defense selectively in people with low basal β-carotene levels owing to nutritional or genetic traits. Thus, we could assume that the small benefit observed in some studies could represent a larger beneficial effect in a small fraction of the studied population depleted in β-carotene and having impaired cognitive function. This β-carotene depletion could be due to genetic or nutritional reasons. Considering both genetic traits and β-carotene serum levels with a personalized nutritional approach tailored to individuals showing deficiency of β-carotene, the effect of this carotenoid on cognition may be much greater. 

Further randomized clinical trials (double-blind and placebo-controlled, considered the “gold standard” of clinical studies) assessing β-carotene and its influence on cognitive function are deserved. Of particular interest would be studies in adults aged 60–70 years, since cognitive decline is a common phenomenon that often accompanies aging. Studying this age group would allow researchers to target a population where cognitive concerns are more prevalent. Such studies should contemplate measurements of serum β-carotene at baseline and a battery of cognitive tests validated at various intervals.

## 5. Conclusions

This is the first systematic review that evaluates the relationship between β-carotene and cognitive function. Most epidemiological evidence supports β-carotene having a positive effect through dietary consumption and vegetable and fruit intake. Some studies have suggested that combining β-carotene with other nutrients with antioxidant properties, such as vitamin E, vitamin C, zinc, or selenium, may have a greater impact on cognitive function when serum levels of it are not depleted.

## 6. Strength and Limitation

This systematic review had a particular strength in its study selection process, assisted by an automated cloud platform. Additionally, the literature search was conducted using a predefined search strategy across multiple databases. Chances for pooling data for meta-analysis were explored but limited because of heterogeneity among the included studies in terms of participant characteristics, dosage and the duration of supplementation, adjustment covariates, and the cognitive assessment tools employed. As for other limitations, the reliability and authenticity of the results may be influenced by the timing of the questionnaires used to evaluate β-carotene intake, which may not accurately reflect long-term dietary habits. The results may also be affected by the use of self-reported data, which could involve inherent social bias. Finally, the measurement of β-carotene in serum only captures a portion of its content, disregarding its presence in body fat and thereby potentially compromising the accuracy of the results.

## Figures and Tables

**Table 3:** List of papers clustered in Topic 2.

Study	Methods	Results	Reference
Zhong et al. (2023)	Dietary intake	Q1 of BC vs. Q4 had lower risks of both CERAD WL decline [odds ratio (OR) = 0.63, 95% confidence interval (CI): 0.44–0.90] and AFT decline (OR = 0.66, 95% CI: 0.47–0.94). Q3 of BC dietary intake had a significantly decreased risk of lower DSST scores (OR = 0.67, 95% CI: 0.48–0.83). For males, dietary BC intake was associated with a decreased risk of AFT decline (OR = 0.51, 95% CI: 0.30–0.85). For females, dietary BC intake was associated with decreased risks of CERAD WL and AFT decline (OR = 0.37, 95% CI: 0.21–0.64; OR = 0.58, 95% CI: 0.37–0.91, respectively)	[29]
Yuan et al. (2020)	Dietary intake	Long-term intakes of total BC and dietary BC were each associated with lower odds of moderate and poor SCF (OR = 0.77 in Q5; *p* < 0.001)	[31]
Beydoun et al. (2020)	Dietary intake	BC intake was associated with a faster decline and poorer performance on the CDT	[30]
Gerger et al. (2019)	Serum levels	Positive correlation of BC serum concentration with verbal memory assessed through the ISLT (*p* < 0.01)	[32]
Nooyens et al. (2015)	Dietary intake	No association between BC intake and cognitive decline	[34]
Johnson et al. (2013)	Serum levels	BC serum concentrations positively correlated with most measures of better cognitive function (*p* < 0.05)	[35]
Péneau et al. (2011)	Dietary intake of BC-rich FVs	The intake of BC-rich FVs was negatively associated with executive functioning scores (*p* = 0.02)	[38]
Akbaraly et al. (2007)	Serum levels	No associations were found between lower BC serum levels and cognitive impairment	[36]
Perkins et al. (1999)	Serum levels	Decreasing serum levels of BC were not associated with poor memory performance	[43]
Perrig et al. (1997)	Serum levels	BC serum concentration (β0.106, *p* = 0.035) remained a significant predictor of semantic memory performance	[28]

Q, quartile. Other abbreviations are presented in the Abbreviations section.

**Table 4:** List of papers clustered in Topic 1.

Study	Supplementation	Results	Reference
Li et al. (2015)	Daily VE (200 mg) and VC (300 mg) combined with BC at 16.7 (group A), 8.4 (group B), 5.6 (group C), or 0 mg/day (group D) or VE alone (5 mg) (group E) for 16 weeks	MMSE scores in A and B were 23.49 ± 4.40 and 23.44 ± 3.62, respectively, significantly higher compared to E (22.32 ± 4.23; *p* < 0.05). HDS scores in A and B were 22.46 ± 4.96 and 21.38 ± 3.97, respectively, significantly higher than the corresponding scores prior to the treatment (18.68 ± 5.77 for A and 19.75 ± 5.46 for B; *p* < 0.05). HDS scores in A and B (22.46 ± 4.96 and 21.38 ± 3.97; *p* < 0.05) were significantly higher compared to E (18.87 ± 4.70; *p* < 0.05).	[33]
Kesse-Guyot et al. (2011)	Daily VC (120 mg), BC (6 mg), VE (30 mg), selenium (100 μg), and zinc (20 mg) in combination or placebo for 8 y	Subjects receiving active antioxidant supplementation had better episodic memory scores (mean difference: 0.61; 95% CI: 0.02, 1.20).	[37]
Kang et al. (2009)	BC (50 mg) every other day or placebo, either alone or combined with VE every other day (402 mg), VC daily (500 mg), or both, for 3.5 y	They found that BC supplements were beneficial among those with low dietary intakes of total carotenoids but not among those with higher intakes (*p* for interaction = 0.02).	[42]
Grodstein et al. (2007)	BC 50 mg every other day for 18 y	Improvement in global cognitive score (*p* = 0.03), verbal memory (*p* = 0.007), and TICS score (*p* = 0.04).	[40]
Wolters et al. (2005)	Multivitamin capsule (9 mg/d BC) for 6 months	No effect on cognitive performance.	[41]
Smith et al. (1999)	12 mg/d BC, 400 mg/d VE, and 500 mg/d VC in combination or placebo for 1 y	There were very few significant differences between the placebo and multivitamin groups.	[39]

Abbreviations are presented in the corresponding list.

## Data Availability

Not applicable.

## Abbreviations

AFT	Animal Fluency Test
BAT	Berliner Amnesie Test
BC	β-carotene
BDNF	Brain-Derived Neurotrophic Factor
BDS	Behavioral Dyscontrol Scale
BTA	Brief Test of Attention
BVRT	Benton Visual Retention Test
CERAD WL	Consortium to Establish a Registry for Alzheimer’s Disease Word Learning
CFQ score	Cognitive Failures Questionnaire
CDT	Clock Drawing Test
CFT	Category Fluency Test
CVLT	California Verbal Learning Test
COWAT	Controlled Oral Word Association Test
DCS	Doetinchem Cohort Study
DFR	Delayed Free Recall
DSST	Digit Symbol Substitution Test
EBMT	East Boston Memory Test
FDST	Forward digit span test
FFQ	Food Frequency Questionnaire
FOME	Fuld Object Memory Evaluation
FVs	Fruit and vegetables
GDRS	Global Deterioration Rating Scale
HDS	Hasegawa Dementia Scale
ISLT	International Shopping List Task
RI-48	Cued Recall Test
KAI	Kurztest fuer Allgemeine Intelligenz
LDA	Latent Dirichlet Allocation
MMSE	Mini-mental state examination
MoCA	Montreal Cognitive Assessment
NART	National Adult Reading Test
NIH	National Institutes of Health
ONB	One Back Task
PHS	Physicians’ Health Study
PHSII	Physicians’ Health Study II
SIB	Severe Impairment Battery
NHANES	National Health and Nutrition Examination Survey
NHS	Nurses’ Health Study
SCF	Subjective Cognitive Function
TICS	Telephone Interview of Cognitive Status
TMT	Trail Making Test
TMTA	Trail Making Test A
TMTB	Trail Making Test B
VA	Vitamin A
VE	Vitamin E
VC	Vitamin C
WAIS-III	Wechsler Adult Intelligence Scale Revised
WFT	Word Fluency Test
WACS	Women’s Antioxidant Cardiovascular Study
